# Matrix Metalloproteinase-9/Neutrophil Gelatinase-Associated Lipocalin Complex Activity in Human Glioma Samples Predicts Tumor Presence and Clinical Prognosis

**DOI:** 10.1155/2015/138974

**Published:** 2015-11-18

**Authors:** Ming-Fa Liu, Yong-Yang Hu, Tao Jin, Ke Xu, Shao-Hong Wang, Guang-Zhou Du, Bing-Li Wu, Li-Yan Li, Li-Yan Xu, En-Min Li, Hai-Xiong Xu

**Affiliations:** ^1^Department of Neurosurgery, Affiliated Shantou Hospital of Sun Yat-sen University, Shantou 515041, China; ^2^Department of Pathology, Affiliated Shantou Hospital of Sun Yat-sen University, Shantou 515041, China; ^3^Department of Radiology, Affiliated Shantou Hospital of Sun Yat-sen University, Shantou 515041, China; ^4^Department of Biochemistry and Molecular Biology, Shantou University Medical College, Shantou 515041, China; ^5^Institute of Oncologic Pathology, Shantou University Medical College, Shantou 515041, China

## Abstract

Matrix metalloproteinase-9/neutrophil gelatinase-associated lipocalin (MMP-9/NGAL) complex activity is elevated in brain tumors and may serve as a molecular marker for brain tumors. However, the relationship between MMP-9/NGAL activity in brain tumors and patient prognosis and treatment response remains unclear. Here, we compared the clinical characteristics of glioma patients with the MMP-9/NGAL activity measured in their respective tumor and urine samples. Using gelatin zymography assays, we found that MMP-9/NGAL activity was significantly increased in tumor tissues (TT) and preoperative urine samples (Preop-1d urine). Activity was reduced by seven days after surgery (Postop-1w urine) and elevated again in cases of tumor recurrence. The MMP-9/NGAL status correlated well with MRI-based tumor assessments. These findings suggest that MMP-9/NGAL activity could be a novel marker to detect gliomas and predict the clinical outcome of patients.

## 1. Introduction

Glioblastoma multiforme (glioma) is the most common primary brain tumor diagnosed in adults. Despite advances in radiotherapy and chemotherapy, the mortality rate of glioma remains very high, with the majority of patients surviving for a year or less after diagnosis [1]. A major contributor to this poor prognosis is the highly invasive nature of glioma tumors. These statistics illustrate the need for innovative tools to improve the glioma patient outcome. In particular, it is important to easily identify novel or recurrent disease and monitor tumors to assess patient response to treatment.

Matrix metalloproteases (MMPs) are a family of zinc-dependent extracellular endopeptidase enzymes that is involved in the degradation of extracellular matrix and basement membrane [2, 3]. Among the MMPs family, MMP-9 plays an important role in tumor invasion and metastasis [4–6]. Neutrophil gelatinase-associated lipocalin (NGAL) is a member of the lipocalin superfamily [7] that can promote MMP-9 activity in part by protecting MMP-9 from autolysis [7, 8]. NGAL can promote MMP-9 activity by forming a complex with the protease (MMP-9/NGAL), and these complexes appear to be elevated in the tumor samples as well as urine samples from cancer patients, suggesting the formation of the MMP-9/NGAL complex may play a role in tumor progression [9–12]. In particular, MMP-9/NGAL levels are elevated in tumor and urine samples of brain tumor patients, with a positive correlation between MMP-9/NGAL levels in the brain tumor and those in the urine [13, 14].

Despite the findings of elevated MMP-9/NGAL levels in brain tumors, little is known about the relationship between the activity of MMP-9/NGAL in glioma patient response and prognosis. In particular, the significance of preoperative and postoperative urine levels of MMP-9/NGAL with respect to clinicopathological features and clinical prognosis of patients with glioma remains unclear. Due to its association with tumor invasion, we hypothesize that MMP-9/NGAL activity levels in urine could serve as marker of glioma tumor progression and patient prognosis before, during, and after therapy. Therefore, we have measured MMP-9/NGAL activity in glioma tissue and urine samples collected before and after surgery of glioma patient to assess the relationship of MMP-9/NGAL activity levels and disease progression and therapeutic response.

## 2. Materials and Methods

### 2.1. Patients

This study was approved by the Ethics Committee of the Affiliated Shantou Hospital of Sun Yat-sen University (Shantou, Guangdong, China). All patients provided informed consent to participate in the study. All glioma patients had tumors that were detectable by Magnetic Resonance Imaging (MRI) at time of urine specimen collection. All patients underwent surgery to remove the tumor between 2010 and 2012. The presence of brain tumors was further confirmed by pathologic analysis, and tumor grade was evaluated according to World Health Organization (WHO) classifications by two pathologists. No patients were currently receiving chemotherapy or radiotherapy at the time of sample collection, and no patients had known systemic inflammatory disease, trauma, vascular malformations, or nonbrain tumors. Thirty-five glioma patients were enrolled in this study, with a median age of 49 years (range: 5–65). The 28 control subjects were healthy, age- and sex-matched volunteers, with a median age of 47 years (range: 16–65). Paraffin and snap-frozen sections of nonneoplastic brain tissues from 8 patients with intractable epilepsy were also included as controls. Patients who died of diseases not directly related to their gliomas, or due to unexpected events, were excluded from this study. There were no statistically significant differences in age or sex between tumor and control groups.

### 2.2. Collection of Tumor and Urine Samples and MRI Scans

Glioma tumor tissue and adjacent nontumor tissue (1 cm from the tumor margin) were collected during tumor resection surgery [15]. Fresh urine samples were collected in the morning one day prior to surgery (Preop-1d urine) [9, 16], the day after surgery (Postop-1d urine), one week after surgery (Postop-1w urine), and on the day of tumor recurrence diagnosis (Post-*r* urine). For each patient, all tumor and urine samples were collected by the same collector according to the above described schedule. All the specimens were snap-frozen in liquid nitrogen and stored at −80°C for zymography analysis as described below. On each collection day, patient brains were imaged by MRI to assess tumor size. The initial tumor size on MRI scans on the day before operation was verified and measured by two radiologists [17, 18].

### 2.3. Gelatin Zymography

Urine and tissue samples were submitted to gelatin zymography as described previously [14, 16], with some modifications. Urine samples were flash-frozen immediately after collection and stored at −80°C until assay. Aliquots of each sample were centrifuged at 4,000 rpm for 5 minutes (min) at 4°C and the supernatants were collected. Urine samples (10 *μ*L) were mixed with buffer consisting of 2% SDS, 0.1 M Tris (pH 6.8), 20% glycerol, and 0.02% bromophenol blue. The same lysis protocol was followed to prepare tissue extracts from tumor and adjacent nontumor tissue.

Gelatin zymography was performed as described previously [19, 20]. Briefly, tissue extracts and urine were subjected to nondenaturing SDS-PAGE through 10% polyacrylamide gels containing 0.1% gelatin. Gels were rinsed in washing buffer (50 mM Tris-HCl, pH 7.5, 2.5% Triton X-100) at room temperature for 1 hour (h) and incubated overnight at 37°C in incubation buffer (50 mM Tris-HCl, pH 7.5, 10 mM CaCl_2_, and 150 mM NaCl). Gels were fixed in 50% methanol and 10% acetic acid and stained with 0.1% Coomassie blue R250. After destaining in 5% methanol and 7.5% acetic acid, proteolytic degradation of gelatin was visualised as a clear band against a blue background of stained gelatin. Proteolytic signals were quantified by densitometry using the AlphaVIEW SA image analysis system (ProteinSimple, Santa Clara, CA, USA); the relative densitometric units were obtained for statistical analysis.

### 2.4. Statistical Analysis

Statistical analysis was carried out using SPSS version 13.0 software for Windows (SPSS Inc., Chicago, IL, USA). The association between MMP-9/NGAL activity in Preop-1d urine of glioma patients and urine of control subjects was analyzed using a chi-square test. The nonparametric Spearman rank correlation coefficient was used to assess the significance of the relationship between MMP-9/NGAL activity in glioma tumor tissue and that in urine samples. The relationship between MMP-9/NGAL activities with clinicopathological characteristics of glioma patients was analyzed using a nonparametric Mann-Whitney *U* test. For all analyses, a two-tailed *p* value of less than 0.05 was considered statistically significant.

## 3. Results

### 3.1. High MMP-9/NGAL Activity in Tumor and Urine Samples of Glioma Patients

**Figure 1:**
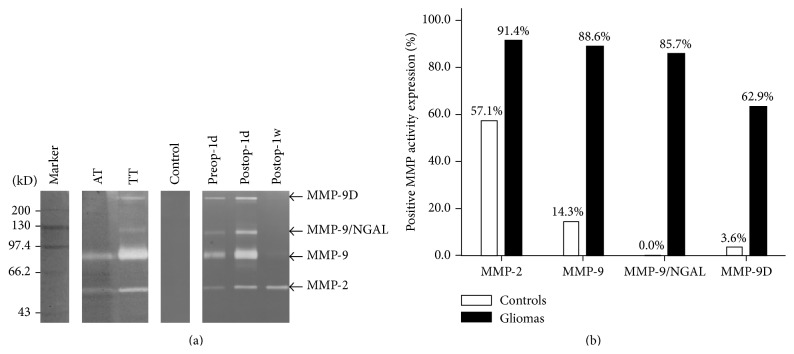
Enzymic activity of MMPs in tumor and urine samples of glioma patients. (a) Gelatin zymography analysis of specimens from a representative control subject and a patient with glioma. Arrows indicate the four active MMPs detected in TT samples (MMP-2, MMP-9, MMP-9/NGAL, and MMP-9D), whereas only weaker MMP-2 and MMP-9 are present in AT from the same patient. Urine from the control had no detectable MMP activity band but had visible MMP activity in Preop-1d urine. MMP-9, MMP-9/NGAL, and MMP-9D activities in Postop-1d urine were significantly increased and quickly decreased in Postop-1w urine, compared with that in Preop-1d urine. (b) Quantification of control and glioma patients with MMP-2, MMP-9, MMP-9/NGAL, and MMP-9D activities detected in Preop-1d urine. Among the 35 tumor patients, MMP-9/NGAL activity was detected in most gliomas (positive for 85.7%); it was not detected in control subjects (AT: adjacent nontumor tissue, TT: tumor tissue, and Preop-1d, Postop-1d, and Postop-1w urine: fresh urine samples were collected in the morning one day prior to surgery, the day after surgery, and one week after surgery).

A total of 21 male and 14 female (a 1.5 : 1 males-to-female ratio) glioma patients were enrolled in this study, 7 patients (20%) were classified as low-grade (grades I and II), and 28 patients (80%) were classified as high-grade gliomas (grades III and IV) (Table 1). MMP activity was evaluated in the tumor tissue as well as preoperative and postoperative urine samples using gelatin zymography. Across the tissue and urine samples, 4 active MMPs were detected (MMP-2, MMP-9, MMP-9/NGAL, and an MMP-9 Dimer); however not all MMPs were present in all patient samples (Figure 1(a)). Adjacent nontumor tissue (AT) and urine of control group did not show MMP-9/NGAL activity, while MMP-2, MMP-9, and trace MMP-9 Dimer activity were detected. In contrast, all four active MMPs, including MMP-9/NGAL, were detected in almost all glioma tissue (TT) and Preop-1d urine of glioma patients, consistent with previous findings [14, 21]. Furthermore, a higher percentage of glioma patients was significantly positive for MMP-9, MMP-9/NGAL, and MMP-9 Dimer activity, compared with control patients (Figure 1(b), *p* < 0.01). MMP-9/NGAL activity was detected in 85.7% of glioma patients and was positively correlated with the activity of MMP-9 and MMP-9 Dimer in Preop-1d urine (Spearman's *r* = 0.83, *r* = 0.74, resp.; *p* < 0.05). However, MMP-9/NGAL activity was not detected in control subjects, suggesting that this complex may be specific to glioma patients. In addition, both MMP-9 and MMP-9/NGAL activity were elevated in high-grade tumors, compared to low-grade tumors (*p* = 0.015, *p* = 0.039, resp.). Moreover, MMP-9, MMP-9/NGAL, and MMP-9 Dimer activity were weak or undetectable in most Postop-1w urine samples (Figure 1(a)), suggesting that removal of the glioma tumor eliminates MMP-9 activity in patient urine samples.

In Preop-1d urine, MMP-9, MMP-9/NGAL, and MMP-9 Dimer activity were positively correlated with the activity detected in the TT samples (Spearman's *r* = 0.658, *r* = 0.793, and *r* = 0.393, resp.; *p* < 0.05). By contrast, MMP-9, MMP-9/NGAL, and MMP-9 Dimer activities in Postop-1d urine were not correlated with their activities detected in TT, Preop-1d, and Postop-1w urine (*p* > 0.05). In Postop-1w urine, only MMP-9/NGAL activity was weakly positively correlated with activity in TT, Preop-1d, and Postop-1d urine (Spearman's *r* = 0.351, *r* = 0.364, and *r* = 0.388, resp.; *p* < 0.05). These data suggest that elevated MMP-9 activity detected in urine samples is strongly correlated with the presence of a primary glioma tumor.

### 3.2. Correlation between MMP-9/NGAL Activity and Clinicopathological Features of Glioma Patients

To assess the relationship between MMP-9/NGAL activity and glioma disease status, we compared MMP-9/NGAL activity and the clinicopathological factors of our patient cohort (Table 2). No significant gender or age related differences were observed between the patient groups (*p* > 0.05). We found that MMP-9/NGAL activity in tumor tissue and Preop-1d urine correlated with the glioma tumor grade and tumor volume (*p* < 0.05); a similar correlation was observed between MMP-9/NGAL activity and the status of astrocytic tumors (*p* < 0.05).

**Figure 2:**
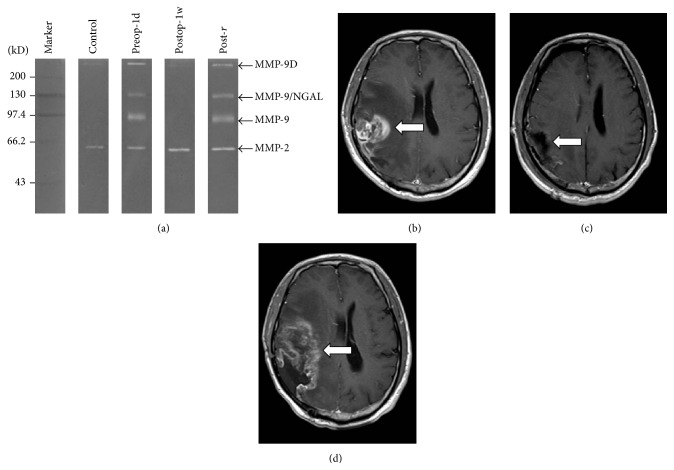
MMP-9, MMP-9/NGAL, and MMP-9D activity is reduced after tumor removal and recovered after tumor relapse. (a) Gelatin zymography analysis of urine samples from a representative subject who has experienced tumor relapse six months after surgery. (b–d) MRI analysis of the tumor (arrow) from the corresponding patient prior to surgery (b), after surgery (c), and at diagnosis of relapse (d) (AT: adjacent nontumor tissue, TT: tumor tissue, and Preop-1d, Postop-1w, and Post-*r* urine: fresh urine samples were collected in the morning one day prior to surgery, one week after surgery, and on the day of tumor recurrence diagnosis).

To investigate the association between MMP-9/NGAL activity and long-term prognosis, including tumor relapse, we assessed patient status one year after surgery. Twenty-eight of the enrolled patients were analyzed by MRI and 14 cases of relapse occurred within 1–6 months after surgery (Figures 2(b)–2(d)). In ten of the relapsing patients (71.4%), MMP-9/NGAL activity in Postop-1d urine was 8-fold higher than the activity in Postop-1w urine, whereas only four patients (28.6%) from the remaining patients groups relapsed (*p* < 0.05). In a limited number of the glioma patients (*n* = 8), urine specimens were collected at the time of relapse diagnosis. In all patients who experienced tumor relapse, the clearance of the urinary MMPs, particularly MMP-9/NGAL in Postop-1w urine, was accompanied by the reappearance of the urinary MMPs in Post-*r* urine samples (Figure 2(a)). Thus, the elevation of urinary MMP-9/NGAL activity after surgery was indicative of tumor relapse in our cohort of glioma patients.

## 4. Discussion

Because of the poor prognosis of glioma, there is an urgent need for an easily detectable biomarker to facilitate early detection of brain tumors. MMP-2 and MMP-9 are overexpressed in experimental glioma models and patient tissue samples and play an important role in glioma progression, particularly in tumor invasion [15, 21–25]. Elevated levels of MMP-2, MMP-9, MMP-9/NGAL, and some high molecular weight MMPs have been found in the urine, cerebrospinal fluid, and tumor tissue specimens from glioma patients [14]. Previous paper found that NGAL and NGAL-Receptor (NGALR) were overexpressed in glioma tissues and significantly associated with poor prognosis and higher tumor grade [26]. Here, we found elevated MMP-9, MMP-9/NGAL, and MMP-9 Dimer activity in both glioma tissues and preoperative urine from glioma patients compared to control subjects. This study demonstrates that urinary MMP-9/NGAL activity levels can predict disease status and prognosis in glioma patients.

MMPs are detectable in the urine of many types of cancer patients [16], and these proteases play roles in both tumor growth and invasion [27]. NGAL was originally purified from human neutrophils and contributes to tumor progression by promoting MMP-9 activity through the formation of a complex, MMP-9/NGAL [8–11, 19, 28–30]. This complex is often elevated in tumors and has been suggested to predict the tumor presence and tumor stage for a variety of cancer types. For example, the MMP-9/NGAL complex activity has been positively correlated with the depth of invasion of esophageal squamous cell carcinoma [19]. Elevated MMP-9/NGAL activity was found in breast cancer patients compared to healthy controls, and serum levels of MMP-9 and NGAL were significantly correlated with and breast disease severity score. These results suggest that the serum measurement of MMP-9 and NGAL may be a useful noninvasive method for monitoring breast cancer progression [12].

Several lines of evidence show that MMP-9 can play direct and indirect roles in glioma tumorigenesis. MMP-9 is detectable in glioma tissues but not in adjacent nontumor tissues [21]. In addition, MMP-9 appears to be involved in the invasion and metastasis of glioma tumors. For example, simultaneous RNAi-mediated depletion of MMP-9 and uPAR significantly reduces the migratory capacity of glioma cells [31]. It is likely that NGAL also promotes glioma progression through its effects on MMP-9; however the specific role of NGAL in glioma tumorigenesis remains unclear.

Although this study utilized a small sample size, and not all patients completed long-term follow-up, we were able to obtain statistically significant and meaningful comparisons between our cohort of glioma patients and matched controls. Furthermore, our findings have the potential to improve glioma therapy and patient monitoring. Specifically, we found that higher MMP-/NGAL activity in both urine and tumor tissue samples was specifically and closely associated with a poor clinical outcome. Further investigation with a larger sample size and urine analysis from additional time points after surgery could more comprehensively evaluate the potential for MMP-9/NGAL to be used as a monitoring tool. Although the precise role of MMP-9/NGAL activity in glioma patients remains unclear, the elevation of MMP-9/NGAL activity in glioma patients and subsequent loss and recovery of activity after surgery and regression suggest that MMP-9/NGAL can serve as useful biomarker to diagnose and predict prognosis of glioma patients.

## Figures and Tables

**Table 1:** Patients and tumor characteristics.

Characteristics	*n* (%)
Gender	
Female	14 (40.0)
Male	21 (60.0)
Median age (range) (years)	
≤50	20 (57.1)
>50	15 (42.9)
Tumor grade	
Low-grade	
I	4 (11.4)
II	3 (8.6)
High-grade	
III	21 (60.0)
IV	7 (20.0)
Tumor classification	
Astrocytomas	28 (80.0)
Oligodendrogliomas	5 (14.2)
Anaplastic ependymoma	1 (2.9)
Central neurocytoma	1 (2.9)
Total	35

**Table 2:** Comparison of MMP-9/NGAL activity in serial samples of glioma to clinicopathological characteristics.

Characteristics	MMP-9/NGAL activity^a^							
	TT	*p*	Preop-1d urine	*p*	Postop-1d urine	*p*	Post-1w urine	*p*
Tumor grade								
Low-grade	5.0	0.027	4.0	0.031	58.5	>0.05	1.0	>0.05
High-grade	20.5		9.5		121.0		7.0	
Astrocytic tumors								
Low-grade	6.5	0.018	3.0	0.025	50.0	>0.05	1.0	0.029
High-grade	21.0		9.0		133.0		7.0	
Tumor size (cm^3^)^b^								
≤100	11.0	0.030	4.0	0.030	49.0	>0.05	4.0	>0.05
>100	30.5		16.0		71.0		10.5	

MMP-9/NGAL activity^a^: the median of relative densitometric units detected by gelatin zymography; Tumor size (cm^3^)^b^: 28 cases high-grade gliomas were measured; ≤100: 13 cases; >100: 15 cases (TT: tumor tissue; Preop-1d, Postop-1d, and Postop-1w urine: fresh urine samples were collected in the morning one day prior to surgery, the day after surgery, and one week after surgery).

## Abbreviations

AT: Adjacent nontumor tissue
MMP-2: Matrix metalloproteinase-2
MMP-9: Matrix metalloproteinase-9
MMP-9/NGAL: Matrix metalloproteinase-9/neutrophil gelatinase-associated lipocalin complex
MMP-9D: Matrix metalloproteinase-9 dimer
MRI: Magnetic Resonance Imaging
NGAL: Neutrophil gelatinase-associated lipocalin
Preop-1d urine: Fresh urine samples were collected in the morning one day prior to surgery
Postop-1d urine: Fresh urine samples were collected in the morning the day after surgery
Postop-1w urine: Fresh urine samples were collected in the morning one week after surgery
Post-*r* urine: Fresh urine samples were collected in the morning on the day of tumor recurrence diagnosis
TT: Tumor tissue.
